# Sun Protection Preferences and Behaviors among Young Adult Males during Maximum Ultraviolet Radiation Exposure Activities

**DOI:** 10.3390/ijerph10083203

**Published:** 2013-07-31

**Authors:** Marilyn Wickenheiser, Mary Kate Baker, Rikki Gaber, Hanz Blatt, June K. Robinson

**Affiliations:** 1 Department of Dermatology, Northwestern University Feinberg School of Medicine, Chicago, IL 60611, USA; E-Mails: mrw77@case.edu (M.W.); rikki-gaber@northwestern.edu (R.G.); h-blatt@northwestern.edu (H.B.); 2 Case Western Reserve University School of Medicine, Cleveland, OH 44106, USA; 3 Department of Community & Behavioral Health, East Tennessee State University College of Public Health, Johnson City, TN 37614, USA; E-Mail: baker.marykate@gmail.com

**Keywords:** sun protection, male adolescent attitudes, primary prevention of melanoma

## Abstract

This study explores sun protection attitudes, preferences, and behaviors among young adult males participating in an open-field activity with extreme ultraviolet radiation exposure. Male drum corps members (*n* = 137) responded to survey questions regarding their behavior and willingness to engage in sun protection and barriers to sunscreen usage. A subset of members (*n* = 31) participated in cognitive interviews exploring various sunscreen products and intervention techniques. Participants were knowledgeable about health risks and protection benefits regarding sun exposure. Generally, males had positive attitudes and normative beliefs about using sunscreen. A barrier to sunscreen re-application was lack of adequate time to reapply sunscreen during the open field activity. Males preferred a towelette application method, but were unfamiliar with its efficacy and proper use. Thus, they were more likely to use the more familiar sunscreen spray. To increase sun protection behaviors and lower skin cancer risk for males participating in open-field activities, breaks must be allotted every 2 h and have sufficient time to allow sunscreen application. Future development and research into delivery systems that rapidly and evenly apply sunscreen may help lower exposure in this population.

## 1. Introduction

Ultraviolet radiation (UVR) exposure is a primary risk factor for developing skin cancer [1,2]. Because sunscreen, when used appropriately, decreases the amount of UVR exposure to the skin, its application is recommended as a skin cancer prevention strategy. Other skin cancer prevention strategies include limiting sun exposure, wearing protective clothing, and avoiding indoor tanning devices [3,4]. Proper sun protection can also reduce the incidence of sunburn, skin irritation, and premature skin aging [5,6].

Annual studies performed by the National Institutes of Health show that, among adults, males between the ages of 18 and 24 are at the highest risk of UVR exposure. For the past decade, this group has consistently reported the highest percentage of sunburns, with 50.03% reporting sunburn in 2010. This group was also the least likely group to usually or always protect themselves from the sun by using sunscreen, with only 13.7% of males 18 to 24 years old reporting usually using sunscreen in 2010 [7]. While research to date has primarily focused on the sun protection and tanning behaviors of young adult females, the attitudes and behaviors of males were poorly understood [8].

Additionally, groups of young adults engaging in open-field activities with high risk of UVR exposure do not regularly apply sunscreen. Young adults participating in organized outdoor activities, such as marching bands or athletics, must practice outside during peak sun hours, follow specific uniform requirements that do not allow for protective clothing, and cannot seek shade. They must rely on sunscreen as their primary form of skin cancer prevention. A recent study suggested that 85% of sun-exposed National College Athletic Association (NCAA) athletes reported no sunscreen use in the past seven days [9].

It is known that young adult males, especially those participating in open-field activities, are at high risk of sunburn and that they do not protect themselves adequately with sunscreen, but the reasons for their inadequate protection have not been determined. This study seeks to further explore the attitudes and behaviors of young adult males in an environment of maximum UVR exposure to inform the development of a targeted skin cancer prevention program.

## 2. Experimental Section

### 2.1. Sample

In the summer of 2012, members of two Illinois regional drum and bugle corps (drum corps) were surveyed about sun protection preferences, sun protection behaviors, and willingness to engage in sun protection behaviors. The drum corps is a marching musical group that consists of young adults between the ages of 18 and 21 performing with brass and percussion instruments and a color guard. We selected this group because, like athletes, they practice in an unshaded, open field for long periods of time during peak sun hours without shade, they have set uniform requirements, and they place importance on performance of a particular physical activity. Thus, they rely on the use of sunscreen for sun protection.

The drum corps specifically practices outside for 10–12 h per day, 7 days a week for 3 weeks in May and June prior to a schedule of competitions in the following 2 months. They receive breaks at 4-h intervals for meals, and receive intermittent shorter water breaks. Site 1, in Rockford, IL, consisted of male and female members, while site 2, in Lisle, IL, consisted of only male members. We collected data from a co-ed site and an all-male site to determine if female presence affected male attitudes and behaviors.

The UV index for Chicago, IL, the nearest large city, during June when data were collected, ranged from 8–10. A UV index of 8–10 denotes a very high risk of harm from unprotected sun exposure.

### 2.2. Quantitative Assessment: Survey

#### 2.2.1. Survey Procedures

Participants completed a voluntary, self-administered questionnaire during their dinner break and received a Popsicle upon completion of the survey. The questionnaire included 40 items regarding attitudes about various forms of sun protection, indoor tanning and sunless tanning, number of painful sunburns, sun protection behaviors and demographic information including ethnic background and skin tone. Survey items along with responses are summarized in Table 1, Table 2, Table 3, Table 4, Table 5, Table 6. In prior research, the survey items were shown to predict sun protection behaviors of Midwest adolescents [10,11]. Survey items were the same as or adapted from previously published measures (alpha values ranged from 0.75 for skin tone to 0.85 for sun protective behavior) [12,13,14]. The Institutional Review Board of Northwestern University approved the protocol.

#### 2.2.2. Survey Measures

To obtain demographic information, questions included ethnic background, family history of skin cancer, and skin type (Table 1). To determine barriers to sunscreen use, survey items asked the length of time required to apply sunscreen, ability to reapply sunscreen every two hours, number of breaks during the day, and negative past experiences the person had with sunscreen use (Table 2). To determine attitudes about sun protection, questions probed willingness to try various methods of sun protection including use of various types of sunscreen, reapplication of sunscreen, seeking shade, wearing sun-protective clothing, and willingness to use self-tanning products (Table 3). Questions regarding sun protection behaviors asked how often the participant uses various sun protection measures on a sunny day and a cloudy day, SPF of the sunscreen used, and history of sunburns (Table 3, Table 4). Tanning behaviors were explored by asking about history of indoor tanning use and history of sunless tanning product use (Table 5). Additionally, these questions asked how helpful some strategies to increase sunscreen use would be such as keeping sunscreen in a readily available location on the sideline or supplying large pump dispensers of sunscreen (Table 6).

**Table 1:** Participants Baseline Demographic Characteristics.

Characteristic		Phantom Regiment (*n* = 61)	Cavaliers (*n* = 76)
*Race*			
	White/Caucasian	54 (89)	72 (95)
	Black/African American	0 (0)	1 (1)
	Asian	1 (2)	0 (0)
	Native Hawaiian or other Pacific Islander	0 (0)	0 (0)
	American Indian or Alaska Native	0 (0)	0 (0)
	Multiracial	6 (10)	3 (4)
*Ethnicity*			
	Hispanic	13 (21)	14 (18)
*Skin Type*			
	Very Fair	11 (18)	7 (9)
	Fair	29 (48)	40 (53)
	Golden to Olive	12 (20)	15 (20)
	Light Brown	9 (15)	11 (14)
	Dark Brown	0 (0)	2 (3)
	Very Dark	0 (0)	0 (0)
	No Response	0 (0)	1 (1)
*Positive Family History of Skin Cancer*		18 (30)	15 (20)

**Table 2:** Barriers to sunscreen use.

	Co-ed (*n* = 61)	All Male (*n* = 76)	Total (*n* = 137)
*On average, how long does it take you to apply sunscreen before going outside? ^*			
Less than 5 min	38 (62)	39 (51)	77 (56)
5–10 min	16 (26)	31 (41)	47 (34)
11–15 min	3 (5)	1 (1)	4 (3)
16–20 min	0 (0)	1 (1)	1 (1)
Greater than 20 min	0 (0)	2 (3)	2 (2)
Do not apply sunscreen before going outside	3 (5)	2 (3)	5 (4)
*How able are you to reapply sunscreen every 2 h throughout the day? ^*			
Never able	12 (20)	3 (4)	15 (11)
Rarely able	23 (38)	14 (19)	37 (27)
Neutral	16 (26)	17 (23)	33 (24)
Usually able	10 (16)	25 (33)	35 (26)
Always able	0 (0)	16 (21)	16 (12)
*How many water/sunscreen breaks occur during the drum corps day? ^*			
None	4 (7)	0 (0)	4 (3)
1	5 (8)	1 (1)	6 (4)
2	5 (8)	1 (1)	6 (4)
3	3 (5)	2 (3)	5 (4)
4	4 (7)	1 (1)	5 (4)
5 or more	39 (65)	71 (93)	110 (80)
*Which of the following negative experiences have you had with sunscreen? +*			
Oily/sticky texture interferes with outdoor activity	44 (72)	51 (67)	95 (69)
Sunscreen gets into and burns eyes	31 (51)	46 (61)	77 (56)
Dislike the smell of sunscreen	17 (28)	13 (17)	30 (22)
Sunscreen takes too long to apply	28 (46)	39 (51)	67 (49)

^ One participant did not reply to this question; + participants were allowed to select more than one answer choice.

**Table 3:** Comparison of willingness to engage in sun protection and sun protection behaviors.

		Co-ed (*n* = 61)	All-Male (*n* = 76)
		Mean	Mean
*Willingness to: #*			
	Try various sunscreens to see which I like best	2.92	3.54
	Reapply sunscreen after sweating heavily	3.10	3.47
	Find shade when I am outside between 10 AM and 3 PM	3.57	4.06
	Wear a shirt with sleeves	2.25	2.28
	Wear a sun hat with a brim	3.82	3.49
	Stop trying to tan	2.22	2.66
	Try self-tanning creams	1.48	1.70
	Get a spray tan to see how I look	1.20	1.45
*On a warm, sunny day of drum corps, how often do you: ^*			
	Wear sunscreen	3.77	4.09
	Wear a shirt with sleeves that cover your shoulders	2.52	2.79
	Wear a hat	4.21	4.18
	Wear sunglasses	3.22	4.02^ a^
	Spend time in the sun in order to get tan	2.32	2.45
*On cloudy day of drum corps, how often do you: ^*			
	Put on sunscreen	3.70	3.69
	Wear a shirt with sleeves that cover your shoulders	2.41	2.66
	Wear a hat	4.54	4.12^ b^
	Wear sunglasses	3.05	3.82^ b^
*History of sunburns*			
	Number of sunburns in the last two weeks	1.64	1.75

# Participants responded to a 5-point Likert-type scale defined as follows: 1 = Not at all willing, 2 = Slightly willing, 3 = Somewhat willing, 4 = Very willing, 5 = Extremely willing; between group differences assessed using one-way ANOVA; ^Participants responded to a 5-point Likert-type scale defined as follows: 1 = Never, 2 = Rarely, 3 = Sometimes, 4 = Often, 5 = Always; between group differences assessed using Mann-Whitney U Test; ^a^ statistically significant difference between groups, *p* < 0.01; ^b^ statistically significant difference between groups, *p* < 0.05.

**Table 4:** SPF of sunscreen used.

	Co-ed (*n* = 61)	All Male (*n* = 76)	Total (*n* = 137)
SPF 4 or less	1 (2)	1 (1)	2 (1)
SPF 5–14	0 (0)	0 (0)	0 (0)
SPF 15–29	5 (8)	13 (17)	18 (13)
SPF 30 or greater	53 (87)	62 (82)	115 (84)
Do not know what SPF	0 (0)	0 (0)	0 (0)
Do not wear sunscreen	2 (3)	0 (0)	2 (1)

**Table 5:** Comparison of tanning behaviors.

		Co-ed (*n* = 61)	All Male (*n* = 76)	Total (*n* = 137)
*Have you ever used indoor tanning?*				
	Yes	2 (3)	8 (11)	10 (7)
	No	59 (97)	68 (89)	127 (93)
*Have you ever used sunless tanning products? ^^*				
	Yes	2 (3)	5 (7)	7 (6)
	No	57 (97)	71 (93)	128 (94)

^^ Two participants did not respond to this item.

**Table 6:** Likelihood of Strategy Aiding Sunscreen Reapplication.

Item	Never Helpful	Rarely Helpful	Neutral	Often Helpful	Always Helpful
Keeping sunscreen next to my water jug during rehearsal. *	23 (17.2%)	14 (10.4%)	46 (34.3%)	38 (28.4%)	13 (9.7%)
Using a large pump sunscreen dispenser located on the sidelines near my water jug. *	25 (18.7%)	16 (11.9%)	49 (36.6%)	30 (22.4%)	14 (10.4%)

* *n* = 134, 3 participants did not respond to this item.

#### 2.2.3. Quantitative Data Statistical Analysis

Data collected using paper surveys were entered electronically and checked for errors. To compare responses from males in the co-ed group with those from males in the all-male group, we used t-tests and analyses of variance for continuous variables, *Χ*^2^ tests for categorical variables, and Mann-Whitney U tests for ordinal variables. *P*-values less than 0.05 were considered statistically significant. Data analyses were conducted using SPSS Version 20.

Demographic data (Table 1) as well as data regarding indoor and sunless tanning use (Table 5) were used to construct tables to compare the all-male and co-ed group. Tables display the number of participants that indicated each response option as well as the corresponding percentage of the group that they represented in parentheses. To compare responses between the two groups, *Χ*^2^ tests were used.

Data describing barriers to sunscreen use (Table 2) and SPF of sunscreen used (Table 4) were summarized as the number of participants from each site that responded positively for each response option as well as the percentage of the site this number represented. Rather than comparing the responses between the two sites for these survey items, data were examined in such a way as to analyze trends for drum corps as one entity.

Finally, willingness to engage in various forms of sun protection was assessed using a 5-point Likert-type scale (1 = Not at all willing; 5 = Extremely willing). Sun protection behaviors were assessed using a 5-point ordinal scale (1 = Never; 5 = Always). Data were summarized using the mean value for each site (Table 3), and mean values were compared between the all-male and the co-ed site. To compare willingness to engage in sun protection behaviors between the two groups, a one-way ANOVA test was used. To compare actual sun protection behaviors between the two groups, a Mann-Whitney U test was used.

### 2.3. Qualitative Assessment: Cognitive Interviews

#### 2.3.1. Cognitive Interview Procedures

Based on the quantitative analysis of the survey responses, as well as extensive literature review regarding various sun protection intervention strategies, more probative questions were formulated and compiled into a moderator-guided cognitive interview (MGCI). The MGCI was designed to assess potential intervention strategies for adult males participating in organized outdoor activities and to further probe sun protection behaviors, barriers to sunscreen use, normative beliefs about sun protection, and perceptions of sun exposure risk. Normative beliefs refer to the perceived behavioral expectations of important individuals or groups such as family and friends [15].

The first data collection method was to show two pictures of sun damaged faces, one that depicted mottled pigmentation of a young adolescent and another that showed the aging face of an elderly male. The picture of the adolescent was chosen to display immediate consequences of sun damage and because the age of the individual was more salient to the participants. The picture of the elderly male was shown to display the results of sun damage over time. The second data collection method was to use sunscreen application methods that were easy to apply, quickly absorbed, and had a texture that would not interfere with performance of the required activity. We elicited beliefs and preferences regarding five commercially available modes of sunscreen application—spray, lotion, clear gel, wax stick, and towelette. We also noted what characteristics males take into account when choosing a sunscreen. The Institutional Review Board of Northwestern University approved the protocol for the MGCI.

Four interviewers conducted the scripted MCGI with a subset of male participants from the co-ed site (*n* = 31). These participants were selected on a volunteer basis and received pizza, popsicles, and Gatorade for their participation. For fidelity, audio was recorded and transcribed for assessment alongside the summary of the participant responses written by the moderators. A content-analytic procedure was used to assess the responses, which were sorted into generalized categories. There were no differences in the responses provided to the three female interviewers *vs.* the one male interviewer. Because data analyses revealed no significant difference between the two groups, the MGCIs were conducted at one site. The co-ed site was chosen because its tour schedule allowed for data collection to take place at the site where the initial survey was given. This minimized potential confounding variables associated with data collection at a new location.

#### 2.3.2. Qualitative Data Statistical Analysis

In order to analyze participants’ reactions to various sunscreen application methods, a composite score was used. Composite scores were created by averaging participants’ responses to 4 aspects of each sunscreen type (texture, ease of application, scent, and packaging). Reactions were scored from 1 (Do Not Like It) to 3 (Like It). The difference in scores between the two highest scoring application methods, the sunscreen spray and the sunscreen wipe/towelette, neared statistical significance (*t*(29) = 1.97, *p* = 0.058) (Table 7). Additionally, sunscreen preference is summarized as the number of participants who gave each application method a rank (1 = most preferred; 5 = least preferred) as well as the percentage of the sample that number represented (Table 8).

## 3. Results

### 3.1. Sample

A total of 137 male drum corps participants elected to complete the surveys, which was 54.8% of the male drum corps population from both sites. Participants, who ranged in age from 18 to 21, represented 58% of male members at site 1 and 52.4% of members at site 2. The survey was completed on a volunteer basis and reasons for refusal to participate were not elicited. Additionally, 3 participants who failed to correctly complete the survey form were excluded. There were no significant differences in background characteristics between the two locations (Table 1).

### 3.2. Quantitative Assessment: Survey

#### 3.2.1. Sun Protection Behaviors

With respect to sun protection behaviors, most (*i.e.*, 96%) participants reported that they applied sunscreen each morning, with the majority (90.5%) spending less than 10 min on the activity. While participants consistently reported having five or more breaks during the day, only 37.5% reported “*often*” or “*always*” being able to reapply sunscreen every 2 h. Interference with playing an instrument because of sunscreen’s oily texture was the most often cited barrier to sunscreen application (71.4%), followed by sunscreen running into their eyes and burning (57.9%) and sunscreen taking too long to apply (50.4%) (Table 2).

#### 3.2.2. Comparison of Co-Ed and All-Male Site Participant Willingness and Behaviors

In comparing the sun protection willingness and behaviors of male adolescents at the co-ed site with the all-male site, male responders at the all-male site were significantly more likely to report wearing sunglasses (*Χ^2^* (1 df) = 8.60, *p* < 0.01) “*often”* or “*always*” on a warm, sunny day than males at the co-ed site. There was no statistically significant difference in willingness to engage in sun protection between the two groups. Males in the two groups did not differ significantly in the number of sunburns (1.67 burns at the all-male site *vs.* 1.64 burns at the co-ed site) experienced during the 2 weeks prior to the survey (Table 3, Table 4). The two groups did not differ significantly in indoor and sunless tanning behaviors (Table 5).

#### 3.2.3. Strategies to Enhance Sun Protection

Over one third (38.1%) of participants felt that placing sunscreen beside their water bottle on the sidelines was an effective strategy for enhancing sun protection on a day-to-day basis. One third of participants did not view providing pump bottles of sweat resistant sunscreen on the sideline during water breaks as a way to improve sunscreen reapplication (Table 6). Since participants’ parents purchased the sunscreen, having it provided on the sideline was not an inducement. Furthermore, participants felt that there would not be enough time for all drum corps members to access and use the sunscreen pump.

### 3.3. Qualitative Assessment: Cognitive Interviews

Interviews were completed with a smaller sample of males from the co-ed site (*n* = 31). Males were self-selected to participate in the interview. With respect to sun protection behaviors, nearly all (93.5%) interview participants brought sunscreen to training. Over half (51.6%) brought sunscreen lotions, while an additional 19.4% brought sunscreen sprays. The sun protection factor (SPF) was uniformly 30 or higher. Responders transported their personal sunscreen from the dorms to the practice field daily in their field bags. Most participants (71%) applied sunscreen daily in the morning prior to starting practice. Most reported that they would ask someone to help apply sunscreen to places they were unable to reach. The remaining 29% applied sunscreen during water breaks or after they began to feel burned. Sunscreen was reapplied by 74.2% of participants during lunch or dinner breaks (*i.e.*, every 4 to 6 h).

The most frequently cited reasons for using sunscreen were to avoid burning and to prevent skin cancer or skin damage. With regard to appearance, most males felt that tanned skin was a positive consequence of sun exposure. Some mentioned that aging and wrinkles were negative appearance consequences. The most frequently cited reasons for sunscreen avoidance were time constraint, no need on a given day as it was cloudy or the person would not be out long, and an aversion to sunscreen’s texture. Lastly, normative beliefs about sunscreen were generally positive, with the majority of participants feeling sunscreen effectively prevented sunburns and that other drum corps members, and society in general, endorsed the use of sunscreen.

During the presentation and testing of commercially available sunscreen products, males most liked the ease of application of aerosol spray sunscreens; however, when taking all 4 reaction components (texture, ease of application, scent and packaging) together, the sunscreen towelette received the highest evaluations in all 4 categories (Table 7). The spray sunscreen scored the next highest. However when asked to rank the types in order of preference, the sunscreen spray received the highest rankings. (Table 8). In considering the most important criteria in selecting a sunscreen, most males (74.2%) cited the sunscreen’s ease of even application, followed by familiarity with the product, and rapidity of application.

Finally, upon viewing the color photographs of the mottled pigmentation on the adolescent and the sun damaged face of the elderly man, 64.5% of the male adolescents stated that the images would cause them to use sunscreen more.

**Table 7:** Comparison of Sunscreen Application Methods (*n* = 31).

Application Method		Do Not Like It *n* (%)	Do Not Care *n* (%)	Like It *n* (%)
*Sunscreen Spray*				
	Texture	12 (38.7)	8 (25.8)	11 (35.5)
	Ease of Application	0 (0.0)	0 (0.0)	31 (100.0)
	Scent	5 (16.1)	20 (64.5)	6 (19.4)
	Packaging	4 (12.9)	9 (29.0)	18 (58.1)
	Spray Composite	2.36		
*Sunscreen Stick*				
	Texture	10 (32.3)	8 (25.8)	13 (41.9)
	Ease of Application ^	8 (25.8)	5 (16.1)	17 (54.8)
	Scent	3 (9.7)	13 (41.9)	15 (48.4)
	Packaging	6 (19.4)	10 (32.3)	15 (48.4)
	Stick Composite	2.26		
*Sunscreen Lotion*				
	Texture	5 (16.1)	13 (41.9)	13 (41.9)
	Ease of Application	10 (32.3)	9 (29.0)	12 (38.7)
	Scent	2 (6.5)	15 (48.4)	14 (45.2)
	Packaging	1 (3.2)	14 (45.2)	16 (51.6)
	Lotion Composite	2.29		
*Sunscreen Gel*				
	Texture	6 (19.4)	10 (32.3)	15 (48.4)
	Ease of Application	7 (22.6)	9 (29.0)	15 (48.4)
	Scent	12 (38.7)	12 (38.7)	7 (22.6)
	Packaging	2 (6.5)	16 (51.6)	13 (41.9)
	Gel Composite	2.18		
*Sunscreen Wipe/Towelette*				
	Texture	2 (6.5)	6 (19.4)	23 (74.2)
	Ease of Application ^	3 (9.7)	5 (16.1)	22 (71.0)
	Scent	2 (6.5)	12 (38.7)	17 (54.8)
	Packaging	7 (22.6)	8 (25.6)	16 (51.6)
	Towelette Composite	2.53 *		

^ One participant did not respond to this item; * Composite scores were created by averaging participants’ reactions to 4 aspects of each sunscreen type (*i.e.*, texture, ease of application, scent and packaging). Reactions were scored from 1 (Do Not Like It) to 3 (Like It). The difference in scores between the sunscreen spray and the sunscreen wipe/towelette neared statistical significance (*t*(29) = 1.97, *p* = 0.58).

**Table 8:** Preference of Sunscreen Application Method.

Application Method	Preference #				
	1 *n* (%)	2 *n* (%)	3 ^ *n* (%)	4 ^^ *n* (%)	5 ^^ *n* (%)
Sunscreen Spray	10 (32.3)	11 (35.5)	2 (6.5)	3 (9.7)	3 (9.7)
Sunscreen Stick	1 (3.2)	4 (12.9)	8 (25.8)	7 (22.6)	7 (22.6)
Sunscreen Lotion	10 (32.3)	6 (19.4)	7 (22.6)	5 (16.1)	5 (16.1)
Sunscreen Gel	1 (3.2)	7 (22.6)	7 (22.6)	6 (19.4)	6 (19.4)
Sunscreen Wipe/Towelette	9 (29.0)	3 (9.7)	6 (19.4)	8 (25.8)	8 (25.8)

^ 1, ^^ 2 participants did not respond to this item; # 1 is liked best, 5 is liked least.

## 4. Discussion

Drum corps members were aware of sun protection and the risks of sun exposure. Most members applied sunscreen each morning; however, many still reported sunburns. We speculate that this is because they did not reapply sunscreen frequently enough. Additionally, males gave sunscreen towelettes the best scores for texture, scent, ease of application and packaging; however, they chose the sunscreen spray as their highest overall choice. This suggests they used different criteria to determine which product they preferred.

While drum corps members received five or more breaks throughout the day and many members kept sunscreen on the field, they were unable to reapply every 2 h. Only 37.5% said they were often or always able to reapply with this frequency. While not directly tested, many of the interview participants noted that the water break did not allow enough time to get water and reapply sunscreen. In addition to keeping sunscreen on the sidelines, the water break would need to be extended to allow for reapplication of sunscreen. 

The need for frequent reapplication is not simply to replace sunscreen that has worn off with movement and sweating. Rather, it is most important to reapply to achieve a protective initial thickness of sunscreen. The SPF of sunscreen is tested at a thickness of 2.00 mg/cm^2^. However, many studies have shown that most users apply much less than this, typically between 0.5 and 1.0 mg/cm^2^ [16,17,18]. Application thickness directly correlates with protection, with most users probably achieving between 20–50% of what is expected from the product label [19]. A recent study suggests that a single reapplication 20 min into a 6-h exposure period results in lower cumulative skin UVR dose at the end of the period than either an initial application only or reapplication at 2–4 h [20], which reinforces the need to apply an adequate thickness early into the sun exposure period.

Males in this study gave the sunscreen towelette the highest reaction scores when assessing its texture, scent, ease of application, and packaging. However, when asked to rank the different types of sunscreen in the order that they would be most likely to use them, the spray ranked the highest. This either means that they may not place high importance on the 4 qualities outlined above, or they may have considered some other factor (s) in their decision-making. Many noted that trust of the product was considered when making this decision. When commenting about the towelette, male participants said they liked the product, however because it was something they had never seen, they did not know how much to apply or if it was effectively working to protect them.

This is a valid concern because effectiveness of protection depends on applying sunscreen to appropriate thickness. Although study participants may prefer the sunscreen spray and the towelette, they may need to apply multiple coats to achieve an adequate layer of sunscreen to be protected. A recent study showed that children using sunscreen in three different forms: pump, squeeze bottle, and roll-on only achieved a coat thickness of 0.75 mg/cm^2^, 0.57 mg/cm^2^ and 0.22 mg/cm^2^, respectively [21], suggesting that form of application influences how much is applied. Male study participants preferred sunscreen forms that were quick to apply, non-greasy, and absorbed quickly, as to not interfere with performance. Education about how to appropriately apply preferable sunscreens may help them to achieve both comfort and protection when participating in outdoor activities.

Males in the all-male corps reported wearing sunglasses significantly more often on a warm, sunny day than males in the mixed gender group. However, the two groups did not differ significantly in willingness to use sun protection. While we had expected female presence to moderate male sun protection behavior, the findings suggest differently. Female presence did not have a significant effect on male sun protection use.

The limitations of this study include the small sample size and possible selection bias. First, within the small sample size there were disproportionately more non-Hispanic Whites than ethnic minorities. Thus, the study included a large proportion of the ethnic group at highest risk for developing UVR-induced skin cancer. Secondly, participation in the study was voluntary and those who chose to participate may have been more concerned about sun protection or health, thus a nonresponse bias may have been present. This study included one unique group of people that are members of drum corps. Further investigation is required to determine whether or not these results are generalizable to athletes or males in general.

## 5. Conclusions

Athletes and young adults engaging in open field activities are at an especially high risk of UVR exposure because their practices take place during peak sun hours without the protection of shade. Due to heat and other uniform requirements, it is not practical for those engaging in open field activities to wear hats or long-sleeved shirts for protection from sun exposure. This group must rely on sunscreen with frequent reapplication as their primary form of sun protection. During open field activities, regular breaks at 2 h intervals need to allow enough time to apply sunscreen, and to get water. Breaks should be more frequent early on in the sun exposure period to apply a thick enough base sunscreen layer. It is estimated that breaks would require approximately 10 min. Physicians could integrate this research into practice by informing young adults about proper sun protection, and working with leaders of organized outdoor activities to allow frequent breaks lasting long enough to provide for adequate sunscreen application. Directors of drum corps and other leaders of outdoor activities should be educated about the need for sunscreen application and reapplication when participating in extended outdoor activities with high levels of UVR exposure.

The use of sunscreen sprays and towelettes saturated with sunscreen needs to be further explored. Instructions for proper application should be developed to increase efficacy of use. Sunscreen sprays are currently included in the Food and Drug Administration (FDA) monograph as safe and effective forms of sunscreen application, although there is some concern of inhalation hazard with use. Additionally, towelettes are not included as part of the FDA monograph at this point in time. Recommendations for this age group will be dependent on further investigation of these sunscreen application methods [22].

The attitudes and behaviors of males in this study can inform the study of male athletes and others who engage in open field activities with the intention of developing of a targeted skin cancer prevention program.
